# 혈액투석 환자에서 동정맥루 천자 방향 및 방법에 따른 재순환율과 투석 적절도의 차이: 단일집단 사전-사후 연구

**DOI:** 10.7586/jkbns.25.012

**Published:** 2025-05-22

**Authors:** WooJeong Ban, YulHa Min, Jungmin Lee, Soo-Hyun Nam

**Affiliations:** 1 College of Nursing, Kangwon National University, Chuncheon, Korea; 1 강원대학교 간호대학; 2 Department of Nursing, Hallym University, Chuncheon, Korea; 2 한림대학교 간호학부; 3 Department of Nursing, GyeongKuk National University, Andong, Korea; 3 국립경국대학교 간호학부

**Keywords:** Kidney failure, chronic, Renal dialysis, Kideney, artificial, Arteriovenous fistula, Cannulation, 만성 신부전, 신장 투석, 인공신장, 동정맥루, 캐뉼라 삽입

## Abstract

**Purpose:**

This study aimed to determine whether the direction of arterial needle insertion and the bevel orientation during arteriovenous fistula (AVF) cannulation affect the recirculation rate and dialysis adequacy, thereby providing evidence relevant to dialysis nursing care. A single-group pretest-posttest design was employed.

**Methods:**

This study included 54 hemodialysis patients at Hallym University Sacred Heart Hospital from July 24 to September 16, 2023. Eligibility criteria included age ≥ 18, AVF use for ≥ 3 months, no recent vascular interventions, and AVF blood flow ≥ 600 mL/min. The recirculation rate was measured with an HD03 monitor at 2–2.5 hours post-initiation, and dialysis adequacy was assessed via Kt/V. The paired t-test was used for analysis.

**Results:**

Among 54 participants, 57.4% were male, and 57.4% had been receiving hemodialysis for ≤ 5 years. The mean AVF blood flow was 982.52 mL/min. No significant differences in the recirculation rate or dialysis adequacy were found based on needle direction or bevel orientation.

**Conclusion:**

The findings suggest that arterial needle direction and bevel orientation during AVF cannulation do not significantly impact the recirculation rate or dialysis adequacy. These results support the standardization of AVF cannulation techniques, which would not pose concerns for altering dialysis efficiency. Further studies with larger samples are recommended.

## 서론

### 1. 연구의 필요성

말기 콩팥병(end-stage renal disease; ESRD) 환자는 신장이식을 받지 않는 한 주기적으로 체내 노폐물을 걸러내어 증상을 완화하는 혈액투석을 평생 받아야 한다[1]. 국내에서 ESRD로 혈액투석을 받는 환자는 2011년 63,341명에서 2023년 110,443명으로 최근 10여 년간 두 배 가까이 증가하였다[2].

혈액투석은 의사의 처방에 따라 투석 시간, 투석액 속도, 혈류 속도, 사용하는 투석막의 종류 등이 결정되며, 투석의 적절성은 다양한 요인에 의해 영향을 받을 수 있다. 건강보험심사평가원에서는 혈액투석 적절성을 평가하기 위해 일정 기간마다 투석 적절도 및 동정맥루(arteriovenous fistula) 상태를 정기적으로 검사할 것을 권장하고 있다[3]. 투석 적절도(dialysis adequacy)는 혈장에서 요소가 제거되는 양을 요소 분포용적으로 나눈 비율로 평가되며[4], 혈류 속도의 감소, 동정맥루의 재순환(recirculation), 투석막 이상, 투석 시간 부족 등이 투석 효율성을 저하시킬 수 있다[5].

ESRD 환자의 생존율을 저하시킬 수 있는 주요 요인으로는 ESRD의 원인 질환, 신장 대체요법의 방법, 불충분한 투석, 동반 질환, 심리⋅사회적 요인, 영양 상태 등이 있다[6,7]. 이 중 불충분한 투석은 유지 혈액투석 환자의 생존율을 낮추는 중요한 요인으로 지적되며[8,9], 동정맥루 재순환은 혈액 펌프에 의해 혈류가 350~500 mL/min 이하로 떨어지지 않는 한 발생하지 않지만, 바늘 삽입 오류, 바늘 간격의 과도한 근접, 동정맥루 협착, 혈관 폐색 또는 혈전 발생 시 재순환이 증가하여 투석 효율이 저하될 수 있다[4]. 재순환율이 높을수록 노폐물 제거가 불충분하여 투석 적절도가 낮아지므로[10], 재순환율의 정기적인 평가는 ESRD 환자의 혈액투석 관리에서 중요한 요소로 작용한다[11].

특히 동정맥루 천자 방법(정방향/역방향)과 천자 바늘의 사면 방향이 재순환율과 투석 적절도에 영향을 미친다는 점에서 여러 연구가 진행되었으나, 천자 방향에 따른 효과에 대해 상반된 결과가 보고되고 있다. Kang 등[12]은 정방향 천자의 경우 바늘 축이 짧은 거리를 유지할 경우 재순환율이 증가하여 투석 적절도가 감소한다고 보고하며, 역방향 천자가 재순환율 감소에 유리하다고 주장하였다. 반면, Lee 등[13]은 정방향 천자와 역방향 천자 간 재순환율과 투석 적절도에 유의한 차이가 없다고 보고하며 천자 방향보다는 바늘의 삽입 깊이와 간격이 주요 요인이라고 강조하였다. Harman [14]의 연구에서도 천자 방향보다는 바늘 간격이 5 cm 이상 유지될 경우 재순환율 감소에 더 중요한 영향을 미친다고 제안하였다. 또한 혈관 천자 방향뿐 아니라 바늘 사면의 방향(위/아래)이 재순환율에 영향을 미칠 수 있음을 제시하였으며, 정방향 천자에서 바늘 사면이 위를 향할 때 혈류가 원활하게 유지되면서 재순환율이 감소하는 경향을 관찰하였다. 이처럼 천자 방향과 방법에 관한 연구는 일부 천자 방법이 재순환율에 영향을 미친다는 결과를 보고하지만, 반대로 방향과 무관하다는 연구도 존재하여 명확한 근거를 제시하지 못하고 있다.

현재 국내⋅외에서 천자 방법(정방향/역방향, 바늘 사면 방향 등)에 따른 재순환율과 투석 적절도 비교에 대한 연구는 매우 부족한 실정이다. 따라서 본 연구는 정방향 및 역방향 천자와 바늘 사면 방향(위/아래)에 따른 재순환율 및 투석 적절도를 체계적으로 조사하여, 혈액투석 환자의 근거 중심 간호 중재 방안을 마련하고 투석 효과를 극대화하는 데 기여하고자 한다.

#### 2. 연구 목적

본 연구의 목적은 동정맥루 천자 시 동맥 바늘의 방향과 바늘 사면의 위•아래 방향에 따른 천자 방법이 재순환율과 투석 적절도에 미치는 영향을 확인하는 것이다.

### 연구 방법

#### 1. 연구 설계

본 연구는 단일군 사전 사후 설계를 적용하여 동정맥루 천자 시 동맥 바늘의 방향과 바늘 사면의 위•아래 방향에 따른 천자 방법이 재순환율과 투석 적절도에 미치는 영향을 확인하기 위한 실험연구이다.

#### 2. 연구 대상

본 연구는 2023년 7월 24일부터 9월 16일까지 ESRD를 진단받고 한림대학교 춘천성심병원에 주 3회 혈액투석을 위해 내원하는 환자를 대상으로 다음의 조건을 충족하는 이를 연구 대상으로 하였다.

1) 18세 이상의 성인으로 자발적으로 연구 참여에 동의한 환자

2) 의사소통이 가능하며 정신적, 신체적으로 이상이 없고 설문지의 내용을 이해하여 응답할 수 있는 자

3) 말기 콩팥병을 진단받은 후 자가 동정맥루로 혈관 천자하여 3개월 이상 혈액투석을 받고 있는 자

4) 최근 1개월 이내 Percutaneous Transluminal Angioplasty, Thrombectomy 등 혈관 기능부전과 관련하여 시술을 시행하지 않은 자

5) HD03 Hemodialysis Monitor (Transonic Systems Inc., Ithaca, NY, USA)를 통해 측정된 혈류량이 600 mL/min 이상으로 동정맥루 기능부전이 없는 자 (단, 측정된 혈류량이 600mL/min이하의 경우 투석혈관센터 신장내과 전문의의 자문을 구한 자)

혈액투석 환자의 특성을 고려하였을 때, 노인 환자는 취약계층으로 간주될 가능성이 있어 연구 참여 과정에서 충분한 설명을 제공하고 자발적인 동의를 확보하는 절차를 철저히 준수하였다. 특히, 혈액투석 환자는 장기간의 치료 과정과 신체적 부담이 크기 때문에 연구 참여에 대한 명확한 이해가 필요하며, 이에 따라 연구진은 환자의 상태를 고려한 개별 맞춤형 설명을 제공하였다. 인지 기능이 저하된 경우 보호자 동의를 추가로 받는 절차를 적용하였으며, 연구 참여자의 자율적인 의사결정을 최우선으로 고려하였다. 또한, 취약계층 보호를 위한 추가적인 고려사항을 반영하여 연구 대상자 모집 및 동의 절차를 진행하였다.

연구대상자 수는 G*power 3.1.9 프로그램[15]을 이용하여 paired t-test를 위한 양측 검정, 효과크기 0.5, 유의수준 0.05, 통계적 검정력 0.90로 하였을 때 44명이 산출되었다. 본 연구에서는 탈락률 약 20%를 고려하여 54명을 대상으로 실시하였으며, 중도 탈락자는 없었다.

### 3. 연구 도구

#### 1) 일반적 특성

일반적 특성으로는 혈액투석 환자를 대상으로 한 선행연구[16]를 바탕으로 하여 연령, 성별, 결혼 상태, 직업 등 인구 사회학적 특성과 투석 받은 기간, 주당 투석 횟수, 동반 질환 등의 10문항으로 구성된 질병 관련 특성을 설문지를 통하여 조사하였다. 참가자의 생물학적 성(sex)은 대상자가 출생 시 보고한 정보를 기준으로 결정하였다. 수기로 설문지 작성이 어려운 경우에는 연구자가 설문의 내용을 직접 읽어주고 답변을 기재하도록 하였다.

#### 2) 혈액투석 처방

1회 4시간씩, 주 3회의 표준 투석 처방에 의해 선행된 투석 적절도의 결과와 환자의 건체중, 동정맥루 등 전반적인 상태를 고려하여 처방되었다. 투석 기계는 Fresenius Medical Care Hemodialysis Machine (5008S, Fresenius Medical Care, Bad Homburg, Germany), 투석막은 FX80 Classix (Fresenius Medical Care, Bad Homburg, Germany), 투석 기계의 혈류 속도(blood flow)는 250-300 mL/min, 투석액 관류 속도는 전 환자에서 500 mL/min로 고정하였다. 항응고제는 신장내과 전문의의 처방을 확인 후 대상자의 임상 상태를 고려하여 진행하였다.

#### 3) 재순환율

환자의 동정맥루를 천자한 후 Transonic Hemodialysis Monitor (HD03)를 사용하여 동정맥루 재순환율을 측정하였다. 측정 방법은 동맥 및 정맥 라인에 각각의 초음파 감지기를 부착한 후, 투석기 혈류 방향으로 정맥 라인의 주사 투입구를 이용하여 생리식염수 10cc를 5∼6초에 걸쳐서 주입하면 기기에 자동으로 재순환율이 측정되어 즉시 재순환율의 여부를 판단할 수 있다. HD03의 재순환율 측정 원리는 정맥 라인을 통한 생리식염수 주입으로 혈액 단백질 농도 및 초음파 속도가 감소되는 것을 정맥 라인 초음파 감지기가 감지하고, 이후 동정맥루를 통과한 생리식염수가 동맥 라인을 통해 재순환될 때 동맥 라인에 부착된 감지기에 감지되어 재순환 비율로 측정되는 것이다[17]. 환자의 혈액투석 시간은 대부분 평균 4시간 정도 소요되며 재순환율 측정은 HD03 기계의 경우 특별히 정해진 시간은 없으나 적어도 투석 종료 10분 전에는 측정하도록 권고하고 있다[18]. 투석 시작 후 2시간에서 2시간 30분 경과 시점은 재순환율 및 혈류 안정성이 확보되는 시점으로 판단하여 기존 연구 [12,19,20]에 근거하여 선정하였다. 본 연구에서도 투석 시작 후 2시간에서 2시간 30분이 지난 시점에 훈련된 간호사 2인이 측정하며, 연속적으로 2차례 측정하여 평균치를 낸 자료를 비교하였다.

두 측정자의 일치도를 평가하기 위해 측정값 간 상관분석을 실시하였으며, 측정값 간의 신뢰성을 검증하기 위해 급내 상관계수(Intraclass Correlation Coefficient)를 산출하였다. 이를 통해 측정값 간의 일관성을 확보하고, 연구 결과의 신뢰성을 높이기 위한 노력을 기울였다.

#### 4) 투석 적절도(Kt/V)

투석 적절도 Kt/Vurea는 요소청소율인 K와 투석시간인 t를 체내의 urea의 분포배치(volume of distribution)로 나눈 개념[21,22]으로 대상자에게 동의를 구한 뒤 연구자가 직접 의무기록을 열람하여 건강보험심사평가원에서 제공하는 혈액투석 적절도 검사 자동계산 프로그램을 이용하여 계산한 값으로 측정하였다. Pre blood urea nitrogen (BUN)은 투석 시작 직전 동맥혈관의 주사바늘을 천자한 직후에 채혈, Post BUN은 투석 종료 직전에 저혈류 기법을 사용하여 투석액의 속도를 0으로 한 상태에서 혈류속도를 50mL/min으로 감소시키고 5분 후에 동맥혈관에서 채혈하였다[4,23].

Kt/V = -ln(R-0.008 x t) + (4-3.5 x R) x UF/W

* R (urea reduction ratio) :

post hemodialysis BUN(mg/dL) / pre hemodialysis BUN(mg/dL)

* t : time of hemodialysis (min)

* UF : ultrafiltration

* W: post-hemodialysis weight (kg)

#### 5) 동정맥루 천자

천자 시 사용한 바늘은 나비 바늘 형태로 15 Gauge와 16 Gauge이며, 길이가 짧고 굵으며 주사침 끝에 작은 구멍이 있는 바늘이다. 투석 시 최대의 혈류를 얻을 수 있도록 대상자의 혈관 상태에 따라 바늘 구경 선택하여[24] 2023년 7월 24일부터 9월 16일까지 8주 동안 동일한 것을 사용하였다. 실험 설계에 따라 2주에 1회씩 바늘의 방향과 사면 방향(위로 및 아래로 향한 경우)을 변화시켜 환자당 총 4회의 재순환율을 투석 중에 시행하였으며, 혈관 통로 천자 시 간호사가 실험 설계 주차에 맞게 바늘 사면 방향을 확인하여 천자 하였으며, 혈관 천자 후 바늘 삽입 형태로 위, 아래 방향이 구분되어 있어 확인 후 기록하였다.

천자 기법은 동정맥루 천자 시 National Kidney Foundation Kideney Disease Outcome Quality Initiative (NKF KDOQI) 혈관통로 임상진료 지침[23]에 따라 대상자의 동정맥루를 신체검사 등으로 모니터링 후 로프래더 천자(rope-ladder cannulation)로, 투석 시마다 동맥 및 정맥 바늘의 천자 위치를 투석혈관을 따라 시행하는 것으로 혈관 손상을 줄이기 위해 시행하였다[20,23].

천자 간 거리 간격 측정은 정맥 바늘 끝과 동맥 바늘 끝의 거리를 측정하며, 피부 안에 삽입되어 있는 바늘 끝의 거리를 정확히 측정하기 위하여 바늘의 길이(2.5cm)를 미리 측정하고 바늘 천자 후 바늘 시작 간의 거리를 측정하였다. 정방향 천자 시 역방향 천자에 비해 천자 거리가 짧아지게 되는데, 거리의 차이가 재순환율과 투석 적절도(Kt/V)에 영향을 미칠 수 있으므로[12], NKF KDOQI (2006)의 지침에 따라 바늘 끝과 끝을 5cm 이상 차이가 나도록 시행하였다[10].

### 4. 자료 수집

**Figure 1.**
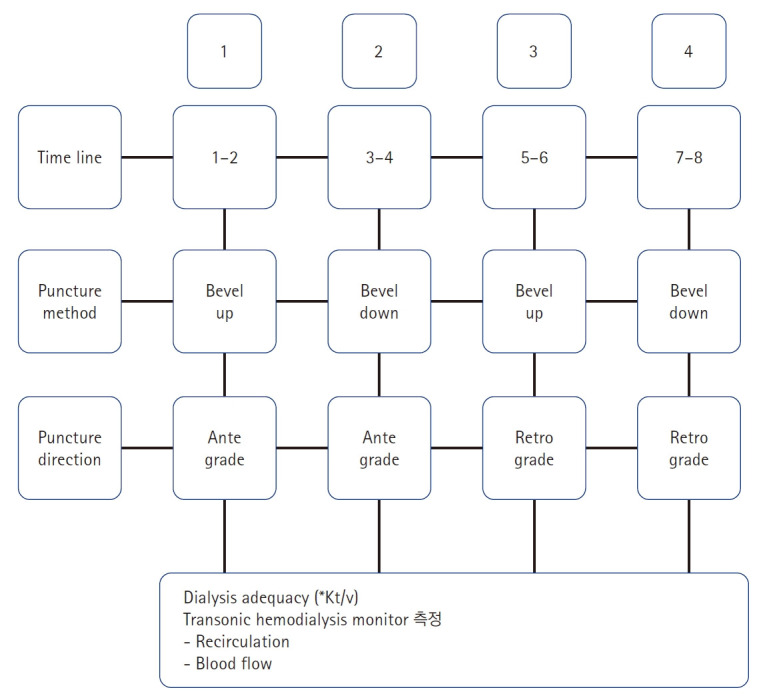
Process of data collection. *Kt/V = K is the urea clearance of the dialyzer (L/hr), t is the dialysis time (hr), and V is the urea distribution volume (L), which closely approximates total body water. It represents the value obtained by multiplying the dialyzer's urea clearance (K) by the dialysis time (t) and dividing the result by the urea distribution volume (V).

연구 대상자의 개인 정보가 노출되지 않도록 임의 코드 번호를 이용하여 익명화된 설문지로 진행하였으며, 참여 의사를 밝힌 대상자가 투석 중인 시간에 연구자가 침상으로 찾아가 안내문을 제공하여 연구의 목적을 설명하고, 동의를 구한 뒤 대상자가 구조화된 설문지를 작성하도록 하였다. 재순환율은 HD03을 사용하여 근무 경력 3년 이상 간호사 2명을 연구보조원으로 선정하였다. 연구의 목적과 기계 사용의 내용을 훈련한 후 측정하였고, 투석 적절도(Kt/V)는 설문지 문항으로 응답이 어려운 대상자는 동의를 구한 뒤 연구자가 직접 의무기록을 열람하여 진행하였다. 설문지 작성에는 약 5-10분정도 소요되었으며, 구체적인 연구 진행 절차는 다음과 같다(Figure 1).

### 5. 자료 분석

수집한 자료는 SPSS version 23.0 (IBM Corp., Armonk, NY, USA)을 사용하여 분석하였다.

1) 대상자의 일반적 특성 및 임상적 특성은 기술통계 방법을 이용하여 실수와 백분율로 구하였다.

2) 천자 방향(정방향과 역방향)과 천자 방법(바늘 사면 위와 아래)에 따른 재순환율 및 투석 적절도(Kt/V)의 차이를 분석하기 위해 대응표본 t-검정(paired t-test)을 실시하였다.

3) 본 연구의 통계적 유의수준은 *p* < .05에서 검정하였다.

### 6. 윤리적 고려

본 연구는 한림대학교 춘천성심병원 연구윤리심의위원회의 승인(N0. CHUNCHEON 2023-06-002-001)을 받아 연구를 진행하였다. 대상자에게 연구의 목적, 소요 시간, 익명성 및 비밀 보장에 대해 설명하고 이해 여부를 확인한 뒤 서면으로 동의서를 받은 후 설문 조사를 시행하였다. 필요 시 연구자에게 언제든지 연락 가능함을 함께 기재하였으며, 연구에 참여를 원치 않거나 참여 중간에 그만두어도 불이익이 없음 등을 알리고 자료를 폐기 요청할 수 있음을 설명하였다. 개인 정보가 노출되지 않도록 임의 코드 번호를 이용하여 익명화된 설문지로 자료수집을 진행하였으며, 작성된 설문지 및 의무 기록은 연구 목적 외에는 사용하지 않으며, 연구가 종료되면 3년간 보관 후 분쇄 폐기할 예정이다.

## 연구 결과

### 1. 일반적 특성 및 임상적 특성

대상자의 나이는 65세 이하가 31명(57.4%)으로 가장 많았으며, 남성이 31명(57.4%), 여성이 23명(42.6%)이었다. 혈액투석 기간은 5년 이하 31명 (57.4%)이 대부분으로 말기 콩팥병의 원인 질환으로 고혈압 22명(40.7%), 당뇨 25명(46.3%), 다낭성신질환 3명(5.6%), 기타 질환이 4명(7.4%) 순으로 나타났다. 동정맥루의 형태는 모두 자가 혈관으로 위치에 따라 구분하며 전완(Radical-Cephalic vein)이 34명(62.9%), 상완(Brachial-Cephalic vein)이 20명(37.1%)으로 나타났다. 혈액투석 처방에 따라 항응고제는 헤파린 초기용량 1500 unit 및 유지용량 500 unit 대상자는 47명(87%), 후탄(nafamostat mesylate) 유지 대상자는 전체 7명(13%) 사용하여 투석 진행하였으며, HD03으로 측정한 혈류량은 982.52 ± 531.36mL/min이었다(Table 1).

### 2. 동맥 바늘 천자 방향에 따른 재순환율 및 투석 적절도의 차이

**Figure 2.**
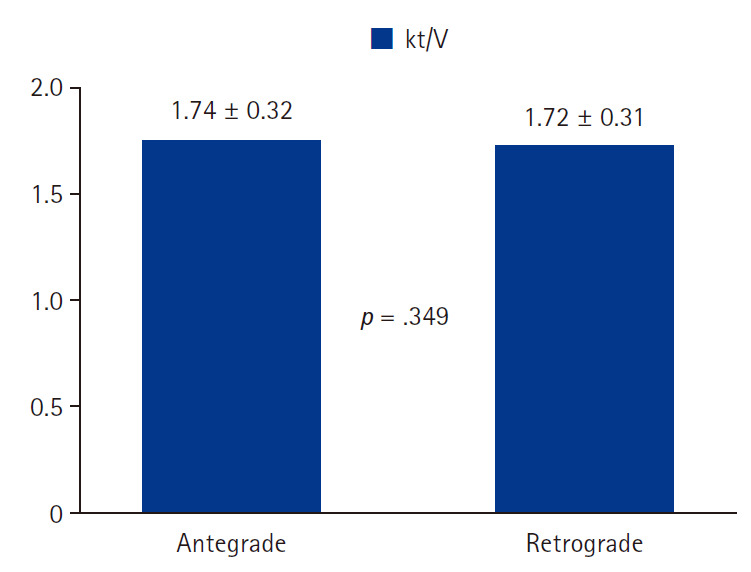
Comparison of Kt/V according to arterial needle direction. Kt/V = K is the urea clearance of the dialyzer (L/hr), t is the dialysis time (hr), and V is the urea distribution volume (L), which closely approximates total body water. It represents the value obtained by multiplying the dialyzer's urea clearance (K) by the dialysis time (t) and dividing the result by the urea distribution volume (V).

**Figure 3.**
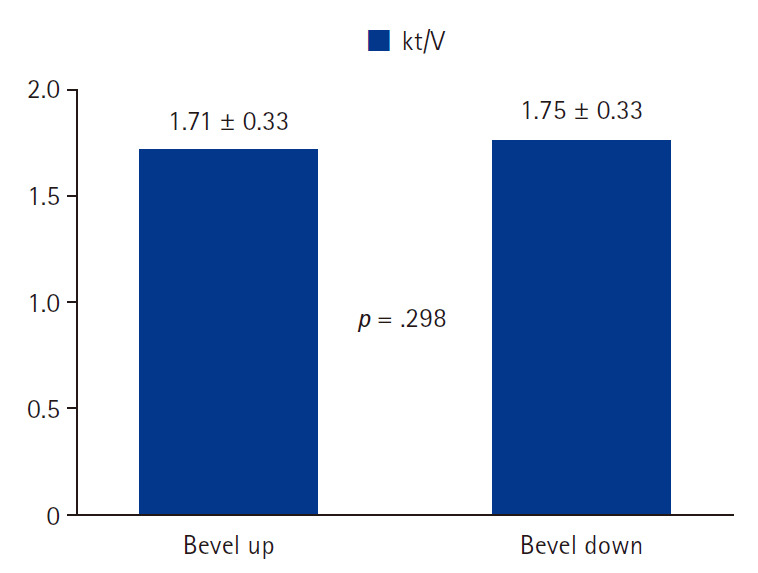
Comparison of Kt/V according to the arterial needle method. Kt/V = K is the urea clearance of the dialyzer (L/hr), t is the dialysis time (hr), and V is the urea distribution volume (L), which closely approximates total body water. It represents the value obtained by multiplying the dialyzer's urea clearance (K) by the dialysis time (t) and dividing the result by the urea distribution volume (V).

투석 시 동맥 바늘의 천자 방향(정방향과 역방향)에 따른 재순환율과 투석 적절도(Kt/V)의 차이를 검증하기 위해 대응표본 t-검정을 실시한 결과는 Table 2와 같다. 천자 방향에 따른 재순환율의 평균 차이는 통계적으로 유의하지 않았다(t = －0.26, *p* = .799). 또한, 투석 적절도(Kt/V)의 평균 차이 역시 통계적으로 유의하지 않은 것으로 나타났다(t = 0.93, *p* = .349) (Figure 2, 3).

### 3. 바늘의 천자 방법에 따른 재순환율 및 투석 적절도의 차이

투석 시 동맥 바늘의 천자 방법에 따른 재순환율과 투석 적절도(Kt/V)의 차이를 검증하기 위해 대응표본 t-검정을 실시한 결과는 Table 2와 같다. 천자 방법에 따른 재순환율의 평균 차이는 통계적으로 유의하지 않았다(t = －0.05, *p* = .963). 또한, 투석 적절도(Kt/V)의 평균 차이 역시 통계적으로 유의하지 않은 것으로 나타났다(t = －1.06, *p* = .298)(Figure 3).

## 논의

본 연구는 혈액투석 환자의 동정맥루 천자 시 동맥 바늘의 방향(정방향과 역방향)과 바늘 사면의 방향(위로 향한 경우와 아래로 향한 경우)이 재순환율과 투석 적절도(Kt/V)에 미치는 영향을 확인하여, 근거 중심의 투석 간호를 제공하는 데 목적이 있다. 동정맥루는 혈액투석 환자의 생명 유지에 필수적인 혈관 통로이지만, 반복적인 천자로 인해 혈관 내막 손상이 발생하고, 이는 혈관 협착 및 폐쇄로 이어질 수 있다[25]. 이러한 문제를 최소화하기 위해 다양한 천자 기법이 임상에서 활용되고 있으나, 특정 천자 방향 및 방법이 투석의 효율성에 미치는 영향을 검증한 연구는 부족한 실정이다. 이에 본 연구는 천자 방향과 방법에 따른 재순환율과 투석 적절도(Kt/V)를 비교하여, 특정 천자 기법이 투석의 효율성을 저해하지 않는지를 확인하고자 하였다.

그 결과, 천자 방향(정방향과 역방향)과 천자 방법(위로 향한 경우와 아래로 향한 경우)에 따른 재순환율 및 투석 적절도(Kt/V)에서 유의한 차이가 확인되지 않았다. 투석 적절도(Kt/V)는 정방향 천자 시 1.74 ± 0.32, 역방향 천자 시 1.72 ± 0.31로 두 군간에 유의한 차이가 없었다(t = 0.937, *p* = .349). 또한 천자 방법에 따른 재순환율과 투석 적절도(Kt/V)를 비교한 결과 재순환율은 없었으며, 바늘 사면 위로 천자 시 투석 적절도(Kt/V)는 1.71 ± 0.33, 바늘 사면 아래로 천자 시 1.75 ± 0.33로 두 군간에 유의한 차이가 없었다(t = －1.062, *p* =.298). 본 연구 결과와 비슷한 결과를 보인 Harman [14]의 연구에서도 동맥 바늘의 정방향 천자 시 재순환율이 통계적으로 유의하게 증가하지 않았다고 하였으며, 천자 간격에 따른 재순환율을 비교한 다른 연구에서 혈관 종류나 천자 간격에 따라 차이가 없었다 [12,13]. 이는 환자 중심의 접근법을 강조하며, 대상자의 혈관 상태나 미용 상의 이유, 편의성, 통증 감소 등의 요인을 반영하여 투석 환자를 대상으로 천자 방향 및 천자 방법, 동정맥루 상태나 요구에 맞추어 천자를 하여도 투석 적절도(Kt/V)가 감소하지 않는다는 것을 의미한다. 즉, 환자가 선호하는 부위에 천자를 시행하거나, 동정맥루 상태 및 개별 요구에 맞춰 천자 방법을 조정하여도 투석의 효과가 저하되지 않음을 시사한다.

본 연구에서 재순환율의 차이가 없는 것은 연구 대상자 모두 동정맥루 기능부전이 없고, HD03을 사용하여 측정한 동정맥루 혈류량이 600 mL/min 이상 유지되는 자가동정맥루를 가진 혈액투석 환자를 대상으로 했기 때문으로 사료된다. 또한 본 연구에서 동맥 바늘과 정맥 바늘 간격을 최소 5 cm 이상 유지하였는데[10], 이러한 관리가 재순환율을 효과적으로 낮추고 안정적인 투석을 가능하게 했을 가능성이 크다.

자가동정맥루의 상태는 투석 기간과 밀접한 관련이 있으며, 장기간 투석을 받을수록 동정맥루의 기능이 저하될 가능성이 높다. 선행연구에 따르면 투석 기간이 10년 이상 경과한 환자에서 동정맥루 협착 위험이 증가하며, 혈류량 감소로 인해 재순환율이 상승할 가능성이 높다고 보고되었다[26,27]. 동정맥루의 기능부전 및 혈류량 감소는 재순환율 증가와 투석 적절도(Kt/V) 저하의 주요 원인으로 보고되고 있다. 따라서 투석 초기 단계에서부터 동정맥루 관리 및 정기적인 혈류량 평가가 필수적이다[28]. Lim 등[20]의 연구에서는 동정맥루 혈류량이 500 mL/min 미만으로 감소한 환자에서 평균 재순환율이 15% 이상 증가하였으며, 이는 혈액 펌프가 혈류를 원활히 순환시키지 못하고 동일한 혈액이 반복적으로 투석기에 의해 여과되는 현상과 관련이 있는 것으로 분석되었다. 또한, KDOQI (2006)에서는 혈관 접근로의 혈류량이 600 mL/min 미만이거나, 1000 mL/min 미만이면서 4개월 전 대비 25% 이상 감소한 경우 혈관 협착 여부를 평가하기 위한 정기적인 영상검사를 권고하였지만[10], KDOQI (2006) 가이드라인 이후 여러 연구 결과를 통해 KDOQI (2019)는 동정맥루에 있어서 혈류장애의 임상 지표를 찾아내기 위해 지식과 경험을 갖춘 의료인이 주기적인 신체검사를 수행하는 것을 권고하여, 혈관통로의 개통성을 개선하고 재순환율 최소화, 투석 적절도를 유지하는데 중요한 역할을 하고 있다[28]. 본 연구는 선행연구 결과를 토대로 동정맥루 혈류량이 600 mL/min 이상 유지되는 환자를 대상으로 연구를 수행하였으며, 대상자의 평균 혈류량이 982.52 ± 531.36 mL/min로 본 연구에서는 재순환율이 측정되지 않았으며, 천자 방향과 바늘 사면 방법의 차이가 재순환율과 투석 적절도(Kt/V)에 미치는 영향이 통계적으로 유의하지 않음을 확인하였다. 또한, 본 연구는 동일한 환자를 대상으로 혈관 천자 시 천자 방향과 방법을 변화시키며 비교하는 단일집단 사전-사후 연구 설계를 적용하여 개별 차이를 최소화하였으며, 보다 신뢰성 있는 데이터를 제공하고자 하였다. 이를 통해 혈액투석 환자의 혈관 천자를 결정할 때, 환자의 개별 혈관 상태와 편의성을 고려한 유연한 접근이 가능함을 시사한다.

인조혈관(Arteriovenous Graft)을 이용한 혈관 접근의 경우, 자가동정맥루(Arteriovenous Fistula, AVF)와 비교하여 협착 및 혈전 발생률이 높다고 알려져 있다[29]. 이는 인조혈관의 내피 세포 형성이 불완전하고, 지속적인 천자로 인해 내벽 손상이 증가하기 때문으로 해석된다. 따라서, 혈액투석 환자의 혈관 접근로 선택 시 AVF가 가능하면 이를 우선적으로 고려하는 것이 바람직하다[21].

한편, 본 연구에서는 15Gauge와 16Gauge 바늘 사용에 따른 차이가 유의하지 않은 것으로 나타났다. 기존 연구에서는 15 Gauge 바늘이 16 Gauge 바늘보다 혈류 속도를 더 높일 수 있지만, 투석 적절도(Kt/V)에는 유의미한 영향을 미치지 않는다고 보고된 바 있다[26]. 이러한 결과는 혈관 상태가 양호한 환자에서는 16 Gauge 바늘을 사용해도 충분한 혈류량을 유지할 수 있음을 의미하며[22], 환자의 혈관 상태와 개인적인 편안함을 고려하여 바늘 크기를 선택할 수 있는 근거를 제공한다.

본 연구에서 투석 적절도(Kt/V)에 유의한 차이가 없었던 이유는 다음과 같이 설명될 수 있다. 첫째, 본 연구 대상자의 동정맥루 혈류량이 모두 600 mL/min 이상으로 유지되는 자가동정맥루를 가진 환자였으며, 동맥 바늘과 정맥 바늘 간격을 최소 5 cm 이상 유지한 점이 투석 적절도(Kt/V)에 영향을 미치는 주요 변수를 효과적으로 관리한 것으로 보인다. 둘째, 재순환율은 투석 적절도(Kt/V) 뿐만 아니라, 투석막의 상태, 혈류 속도, 투석 시간 등 다양한 요인이 투석 적절도(Kt/V)에 복합적으로 작용하기 때문에, 재순환율 차이가 투석 적절도(Kt/V)에 즉각적인 영향을 미치지 않았을 가능성이 있다[4].

결론적으로, 이에 따라 투석실 간호사는 동정맥루 상태를 정기적으로 평가하고, 천자 방법 및 방향을 환자 개별 상태에 맞게 조정함으로써, 환자의 재순환율을 낮추고 투석 적절도(Kt/V)를 유지하며, 동정맥루 합병증 예방에 기여할 수 있다.

본 연구는 다음과 같은 제한점을 가지고 있다.

첫째, 본 연구는 단일집단 사전 사후 설계(one-group pretest-posttest design)로 진행되었다. 이는 동일한 대상자에게 천자 방법과 방향을 변화시켜 재순환율 및 투석 적절도를 측정함으로써, 개별 차이를 최소화하고 보다 신뢰할 수 있는 비교 결과를 얻기 위함이다. 연구 대상자는 HD03으로 혈류량이 600 mL/min 이상인 자가 동정맥루를 가진 환자로 선정되었으며, 연구 기간 동안 동일한 조건에서 투석을 수행하였다. 이를 통해 천자 방법과 방향이 투석 적절도(Kt/V) 및 재순환율에 미치는 영향을 보다 정확하게 평가하고자 하였다. 향후 연구에서는 무작위 대조군 연구(Randomized Controlled Trial)를 적용하여 천자 방법 및 방향의 효과를 보다 객관적으로 검증할 필요가 있다.

둘째, 본 연구는 특정 병원에 내원하는 혈액투석 환자를 대상으로 시행되었으며, 표본 크기가 54명으로 제한적이었다. 연구 대상자의 혈관 상태, 투석 기간, 동반 질환 등 다양한 요인을 반영하지 못했을 가능성이 있다. 따라서 향후 연구에서는 다기관 연구를 통해 보다 다양한 환자군을 포함하고, 혈관 상태 및 개별 특성에 따른 영향을 분석할 필요가 있다.

위와 같은 제한점에도 불구하고, 본 연구는 혈액투석 간호 실무 및 학문적 발전에 다음과 같은 의의를 지닌다. 첫째, 천자 방향 및 바늘 사면의 방향이 재순환율과 투석 적절도에 유의한 영향을 미치지 않는다는 근거를 제시함으로써, 혈액투석실 간호사로 하여금 보다 유연한 천자 기법을 적용할 수 있는 근거를 마련하였다. 본 연구 결과를 바탕으로, 특정 천자 방법을 고수하기보다는 환자의 혈관 상태, 편의성, 통증 감소 등을 고려하여 개별화된 간호를 제공하는 것이 가능함을 시사한다. 더불어, 본 연구의 결과는 혈액투석 간호의 표준화를 위한 근거를 제공한다. 현재 임상에서는 천자 방향과 바늘 사면 방향에 대한 명확한 가이드라인이 부족하며, 일부 시설에서는 특정 천자 방법을 통제하는 경우도 있다. 본 연구는 천자 방향 및 방법에 따른 투석 적절도 차이가 없음을 확인함으로써, 보다 유연한 혈액투석 간호 표준을 확립하는 데 기여할 수 있다. 마지막으로. 본 연구의 결과는 환자 중심적 천자 전략을 적용할 수 있도록 근거를 제공하였다. 일부 환자는 미용적인 이유, 통증 감소, 기존 혈관 손상 등의 이유로 특정 천자 방법을 선호할 수 있다. 동맥 바늘을 천자할 때 상완에 위치한 동정맥루는 혈관의 길이가 짧아 천자 부위 선택에 제약이 따르는데, 본 연구 결과를 바탕으로 보다 넓은 천자 범위를 확보할 수 있는 가능성을 제시한다. 또한, 천자 방향과 방법에 구애받지 않고 환자의 혈관 상태에 맞춰 유연하게 천자를 시행할 경우, 시술자가 매번 불편한 자세로 천자를 해야 하는 부담을 줄일 수 있으며, 시술자의 만족도 또한 향상될 수 있다. 따라서, 임상에서는 다양한 천자 방향과 방법을 적극적으로 적용하여, 개별 환자의 혈관 특성에 맞춘 혈액투석 간호를 제공하는 것이 바람직하다. 본 연구 결과는 이러한 환자 개별 요구를 반영하여 천자 방법을 선택하더라도 투석 적절도가 유지될 수 있음을 확인하였으며, 환자의 만족도 및 치료 순응도뿐만 아니라 시술자의 만족도 또한 향상시키는 데 기여할 수 있다.

## 결론 및 제언

본 연구는 천자 방향 및 천자 방법에 따라 재순환율 및 투석 적절도(Kt/V)에 차이가 있는지 알아보며 투석 간호 표준과 근거 중심 간호를 제공하기 위하여 실시하였다. 본 연구 결과, 천자 방향 및 천자 방법을 각각 다르게 하여 동정맥루 재순환율과 투석 적절도(Kt/V)를 측정한 결과 유의한 차이가 없는 것으로 나타났다. 따라서 환자의 요구도나 개별 혈관상태를 고려하여 정방향과 역방향, 동맥 천자 시 바늘의 사면이 위, 아래로 향하게 천자 방법을 다양하게 적용한다면 잦은 천자로 인한 동정맥루의 손상이나 변형을 예방하여 혈관 내막 증식증을 감소시키고 혈전을 예방하여 더욱 효과적으로 혈관 관리가 가능하리라 사료된다. 특히, 동맥바늘을 천자할 경우 상완에 동정맥루가 있어 사용할 혈관의 길이가 짧아 천자 부위에 제한을 많이 받는 대상자에게 보다 넓은 천자 범위를 확보할 수 있고, 방향에 관계없이 환자 혈관 상태에 따라 혈관 천자를 한다면 매번 부자연스러운 자세로 천자를 실시하는 시술자의 만족도도 높여줄 수 있는 방법이라 사료되므로 임상에서 천자 방향 및 천자 방법을 다양하게 확대하여 혈액투석 간호를 제공할 수 있겠다.

본 연구는 일 대학병원의 자가 동정맥루 환자 중 HD03을 사용하여 측정한 혈류량이 600 mL/min 이상 유지되는 혈액투석 환자를 대상으로 하였으므로 전체 투석 환자를 대표하기에는 한계가 있다. 따라서 본 연구를 기반으로 다음과 같은 제언을 하고자 한다. 자가 동정맥루와 인조혈관은 구조적 차이로 인해 혈류 역학이 다르게 작용할 수 있으며, 이에 따라 천자 방법이 투석 효율성에 미치는 영향도 다를 가능성이 있다. 따라서, 인조혈관을 사용하는 환자군과 자가 동정맥루 환자군을 대상으로 천자 방향과 방법이 재순환율 및 투석 적절도(Kt/V)에 미치는 영향을 비교 분석하는 연구가 필요하다. 또한 본 연구는 혈류량이 600 mL/min 이상인 환자를 대상으로 하였기 때문에, 혈류량이 낮은 환자에게도 동일한 결과가 적용될 수 있는지는 추가 검증이 필요하다. 혈류량이 600 mL/min 이하인 환자에서 천자 방법 및 방향이 투석 적절도(Kt/V)와 재순환율에 미치는 영향을 분석하는 연구가 이루어진다면, 보다 다양한 환자군을 대상으로 한 근거 기반의 투석 간호 지침을 확립하는 데 기여할 수 있을 것이다. 천자 방법은 단순히 투석 적절도(Kt/V)에 영향을 미칠 뿐만 아니라, 환자의 통증 경험과 시술자의 편의성에도 중요한 영향을 줄 수 있다. 따라서, 환자의 주관적인 통증 정도와 시술자의 작업 만족도를 정량적으로 평가하여, 환자와 시술자 모두에게 최적화된 천자 방법을 규명하는 연구가 필요하다. 기존 연구에 따르면 바늘 크기가 혈류 속도에 영향을 미칠 수 있지만, 투석 적절도(Kt/V)에는 유의미한 차이를 보이지 않는 경우도 많다. 본 연구에서도 15 Gauge와 16 Gauge 바늘 사용에 따른 차이가 유의하지 않은 것으로 나타났다. 이에 대한 보다 명확한 근거를 확보하기 위해, 천자 방법(정방향, 역방향)과 바늘 크기(15 Gauge, 16 Gauge 등)에 따른 재순환율 및 투석 적절도를 비교 분석하는 연구가 필요하다.

## Figures and Tables

**Table 1.** General and Clinical Characteristics of the Participants (N = 54)

Variables	Categories	n (%)	M ± SD
Age (yr)	> 65	23 (42.6)	
	≤ 65	31 (57.4)	
Sex	Men	31 (57.4)	
	Women	23 (42.6)	
Cohabitant	Yes	44 (81.5)	
	No	10 (18.5)	
Occupation	Yes	48 (88.9)	
	No	6 (11.1)	
Period of hemodialysis (yr)	> 5	23 (42.6)	
	≤ 5	31 (57.4)	
Causal disease of ESRD	Hypertension	22 (40.7)	
	Diabetes mellitus	25 (46.3)	
	Polycystic kidney disease	3 (5.6)	
	Other	4 (7.4)	
Location of AVF	Forearm	34 (62.9)	
	Upper arm	20 (37.1)	
Anticoagulant drug	Heparin	47 (87.0)	
	Nafamostat mesylate	7 (13.0)	
Blood flow (mL/min)			982.52 ± 531.36

ESRD = End-stage renal disease; AVF = Arteriovenous fistula.

**Table 2.** Differences Depending on Puncture Direction and Puncture Method (N = 54)

Variables	Blood flow		Kt/V	
	M ± SD	t (*p*)	M ± SD	t (*p*)
Puncture direction				
Antegrade	975.73 ± 513.94	‒.26 (.799)	1.74 ± 0.32	0.93 (.349)
Retrograde	988.83 ± 547.46		1.72 ± 0.31	
Puncture method				
Up	981.35 ± 518.30	‒.05 (.963)	1.71 ± 0.33	‒1.06 (.289)
Down	983.74 ± 544.78		1.75 ± 0.33	
Needle size				
15 gauge	948.33 ± 443.05	‒.07 (.941)	1.78 ± 0.32	0.85 (.400)
16 gauge	958.89 ± 589.00		1.72 ± 0.35	

Kt/V = K is the urea clearance of the dialyzer (L/hr), t is the dialysis time (hr), and V is the urea distribution volume (L), which closely approximates total body water. It represents the value obtained by multiplying the dialyzer's urea clearance (K) by the dialysis time (t) and dividing the result by the urea distribution volume (V); M ± SD = Recirculation rates of the arteriovenous fistula were measured using the Transonic Hemodialysis Monitor (HD03) after puncture to assess differences according to puncture direction and method. Data are presented as mean ± standard deviation.

## Data Availability

The data that support the findings of this study are available from the corresponding author upon reasonable request.
